# Modular Deep-Learning Pipelines for Dental Caries Data Streams: A Twin-Cohort Proof-of-Concept

**DOI:** 10.3390/dj13090402

**Published:** 2025-09-02

**Authors:** Ștefan Lucian Burlea, Călin Gheorghe Buzea, Florin Nedeff, Diana Mirilă, Valentin Nedeff, Maricel Agop, Dragoș Ioan Rusu, Laura Elisabeta Checheriță

**Affiliations:** 1 Dentoalveolar Surgery, Faculty of Medicine, University of Medicine and Pharmacy “Grigore T. Popa” Iași, 700115 Iași, Romania; lucianburlea@yahoo.com; 2 National Institute of Research and Development for Technical Physics—IFT Iași, 700050 Iași, Romania; calinb2003@yahoo.com; 3 Clinical Emergency Hospital “Prof. Dr. Nicolae Oblu” Iași, 700309 Iași, Romania; 4 Department of Environmental Engineering, Mechanical Engineering and Agritourism, Faculty of Engineering, “Vasile Alecsandri” University of Bacău, 600115 Bacău, Romania; florin_nedeff@ub.ro (F.N.); vnedeff@ub.ro (V.N.); drusu@ub.ro (D.I.R.); 5 Physics Department, “Gheorghe Asachi” Technical University Iași, 700050 Iași, Romania; m.agop@yahoo.com; 6 Occlusology Estetics and Fixed Prostheses Odonto Parodontology and Fixed Prostheses, Faculty of Medicine, University of Medicine and Pharmacy “Grigore T. Popa” Iași, 700115 Iași, Romania; laura.checherita@umfiasi.ro

**Keywords:** dental caries, oral microbiome, transcriptomics, deep learning, panoramic radiography, twin study

## Abstract

**Background:** Dental caries arise from a multifactorial interplay between microbial dysbiosis, host immune responses, and enamel degradation visible on radiographs. Deep learning excels in image-based caries detection; however, integrative analyses that combine radiographic, microbiome, and transcriptomic data remain rare because public cohorts are seldom aligned. **Objective:** To determine whether three independent deep-learning pipelines—radiographic segmentation, microbiome regression, and transcriptome regression—can be reproducible implemented on non-aligned datasets, and to demonstrate the feasibility of estimating microbiome heritability in a matched twin cohort. **Methods:** (i) A U-Net with ResNet-18 encoder was trained on 100 annotated panoramic radiographs to generate a continuous caries-severity score from a predicted lesion area. (ii) Feed-forward neural networks (FNNs) were trained on supragingival 16S rRNA profiles (81 samples, 750 taxa) and gingival transcriptomes (247 samples, 54,675 probes) using randomly permuted severity scores as synthetic targets to stress-test preprocessing, training, and SHAP-based interpretability. (iii) In 49 monozygotic and 50 dizygotic twin pairs (*n* = 198), Bray–Curtis dissimilarity quantified microbial heritability, and an FNN was trained to predict recorded TotalCaries counts. **Results:** The U-Net achieved IoU = 0.564 (95% CI 0.535–0.594), precision = 0.624 (95% CI 0.583–0.667), recall = 0.877 (95% CI 0.827–0.918), and correlated with manual severity scores (r = 0.62, *p* < 0.01). The synthetic-target FNNs converged consistently but—as intended—showed no predictive power (R^2^ ≈ −0.15 microbiome; −0.18 transcriptome). Twin analysis revealed greater microbiome similarity in monozygotic versus dizygotic pairs (0.475 ± 0.107 vs. 0.557 ± 0.117; *p* = 0.0005) and a modest correlation between salivary features and caries burden (r = 0.25). **Conclusions:** Modular deep-learning pipelines remain computationally robust and interpretable on non-aligned datasets; radiographic severity provides a transferable quantitative anchor. Twin-cohort findings confirm heritable patterns in the oral microbiome and outline a pathway toward future clinical translation once patient-matched multi-omics are available. This framework establishes a scalable, reproducible foundation for integrative caries research.

## 1. Introduction

### 1.1. The Public Health and Clinical Burden of Dental Caries

Dental caries remains one of the most widespread chronic conditions globally, affecting approximately 2.3 billion people and imposing a disproportionate cost burden on low- and middle-income populations [1,2]. Modern consensus no longer views caries as an infectious disease driven by a single pathogen. Instead, it is understood as the clinical outcome of a multifactorial ecological shift: frequent carbohydrate intake drives acidification, disrupting the balance of the supragingival microbial community toward acidogenic and aciduric taxa. As enamel demineralises, inflammatory and matrix-remodelling processes further accelerate tissue breakdown [3,4,5]. Despite decades of public-health messaging and fluoride use, caries incidence remains unacceptably high—partly because current diagnostics rely on visual–tactile inspection and two-dimensional radiography, which are limited to detecting established lesions and offer little prognostic insight into lesion initiation or progression [6]. Adjunctive technologies such as laser fluorescence, near-infrared transillumination, photothermal radiometry and luminescence (PTR-LUM), and ultrasonography have also been explored for early diagnosis and monitoring of caries progression. However, their adoption remains limited due to cost, equipment availability, and variability in sensitivity, reinforcing the need for scalable imaging and computational approaches. In this manuscript, we distinguish between two complementary outcome measures: the radiographic severity score, defined as a continuous estimate of lesion area derived from segmented panoramic radiographs, and the TotalCaries burden, defined as the count of affected teeth recorded in the twin cohort. Severity reflects lesion extent within an image, while burden captures overall disease load at the patient level.

### 1.2. Advances in Microbial and Host Profiling

Over the past decade, 16S rRNA sequencing has provided unprecedented detail into the microbial dimension of caries. Cross-sectional studies consistently show enrichment of *Streptococcus*, *Veillonella*, *Lactobacillus*, and *Actinomyces* in caries-positive plaque relative to healthy controls, while longitudinal work suggests that early “ecological drift” away from health-associated genera precedes detectable demineralisation [7,8,9]. This trajectory, aligned with Takahashi and Nyvad’s ecological theory of caries [10], is inherently polymicrobial: no single taxon fulfills Koch’s postulates, and functional redundancy in fermentative pathways may obscure community-level instability [11]. Twin studies and oral genome-wide association studies (GWAS) further highlight the heritability of the salivary microbiome, showing that monozygotic twins share significantly more similar community structures than dizygotic pairs, even when controlling for environment [12].

On the host side, transcriptomic profiling of gingival and dentinal tissues has revealed complementary molecular shifts. Genes involved in innate immunity, cytokine signaling, and extracellular-matrix turnover—such as *MMP9*, *IL1B*, *IL6*, *TLR2*, and *TLR4*—are repeatedly upregulated at caries-prone sites, emphasizing the immuno-inflammatory dimension of disease progression [13,14,15,16]. Yet few studies have attempted to correlate these molecular changes with contemporaneous imaging or clinical severity metrics, primarily due to the scarcity of patient-matched multi-omic datasets.

### 1.3. The Role of Artificial Intelligence in Dental Imaging and Omics

In parallel, artificial intelligence—particularly convolutional neural networks (CNNs)—has reached expert-level performance in detecting proximal caries, periapical lesions, and periodontal bone loss on bite-wing and panoramic radiographs [17,18,19,20]. Machine-learning approaches have also been applied to classify microbiome and transcriptome data with high accuracy [21], while precision oral-health initiatives such as ZOE 2.0 underscore the need for integrated, child-centred, data-rich pipelines [22]. However, most existing work is image-centric; few models integrate multi-omic data, and even fewer incorporate interpretable deep learning methods that yield biologically actionable insights.

### 1.4. Study Objective and Conceptual Framework

This study addresses these gaps by developing and validating three independent—but harmonised—deep-learning pipelines, each tailored to a distinct data modality: panoramic radiographs, supragingival microbiomes, and gingival transcriptomes. All pipelines are constructed using consistent principles of transparent preprocessing, reproducible training, and model-agnostic explanation. Specifically:

- A U-Net with a ResNet-18 encoder was trained on 100 expert-annotated panoramic images to compute a continuous caries-severity score based on predicted lesion area.
- A feed-forward neural network (FNN) was fitted to 81 supragingival 16S rRNA amplicon sequence-variant (ASV) profiles (750 taxa) following log-transformation, prevalence filtering, and Z-scoring.
- A second FNN was trained on 54,675 probe-level intensities from 247 gingival biopsies, with preprocessing that included log_2_ transformation, quantile normalisation, and feature selection based on variance and correlation with synthetic targets.

Because no patient-matched radiographic severity labels were available for the microbiome and transcriptome datasets, we used random permutations of the imaging-derived severity scores as synthetic targets. These stress tests evaluated the technical stability of preprocessing, model training, and Shapley additive explanations (SHAP) in high-dimensional spaces—while deferring biological interpretation until real patient-matched labels become available.

It is important to note that this is a parallel proof-of-concept across independent datasets, rather than an integrated multimodal analysis of the same patients. Each pipeline was developed and validated within its own cohort, with comparability restricted to methodological robustness and interpretability, not biological integration

### 1.5. Twin-Cohort Validation and Study Layout

Recognizing the continued scarcity of fully aligned multi-modal data, we incorporated an external validation arm: a well-characterised saliva dataset from 49 monozygotic and 50 dizygotic twin pairs [23]. This twin cohort provides two unique advantages:

(i) subject-level alignment between microbial features and real clinical outcomes (TotalCaries count), and
(ii) a natural experiment for assessing microbiome heritability via intra-pair similarity.

Applying the same preprocessing and a deeper FNN architecture (2469 ASVs → 512 → 256 → 128 → 1), we evaluate both predictive accuracy and the degree of genetic influence on salivary community composition.

### 1.6. Paper Structure

The remainder of the manuscript is structured as follows: Section 2 (Materials & Methods) describes dataset acquisition, filtering, normalization, dimensionality reduction, and model configurations, including Bray–Curtis dissimilarity for the twin analysis. Section 3 (Model Architecture) provides design rationale for each pipeline, detailing choices such as dropout regularization and Dice–BCE loss for segmentation. Section 4 (Results) reports segmentation performance (IoU = 0.564 [0.535–0.594]; precision = 0.624 [0.583–0.667]; recall = 0.877 [0.827–0.918]; r = 0.62), summarises FNN cross-validation under synthetic labels (R^2^ < 0; r ≈ 0), and presents twin-cohort findings: significantly lower dissimilarity in monozygotic vs. dizygotic pairs (*p* = 0.0005) and a modest predictive signal for caries (r = 0.253). Section 5 (Discussion) interprets these results, emphasizing that (i) pipeline robustness under label noise is a prerequisite for future patient-matched studies, (ii) host genetics measurably influence the oral microbiome, and (iii) integrated, longitudinal data are now the key barrier to precision dentistry. Section 6 (Conclusion) frames this work as a proof-of-concept, fully documented computational foundation for future multi-omic oral health analytics.

All processed data, trained weights, and the full Google Colab notebook are stored in the authors’ Google Drive. A view-only Colab link is provided for inspection, and the complete archive can be shared by the corresponding author upon reasonable request for non-commercial academic use.

## 2. Materials and Methods

This section describes data acquisition, preprocessing procedures, and the design of independent machine-learning pipelines for radiographic, microbiome, and transcriptomic modelling of dental caries severity. Because the datasets do not share subject identifiers, each modality was analysed independently; cross-modal comparisons are limited to pipeline stability and interpretability.

### 2.1. Data Sources

#### 2.1.1. Supragingival Plaque Microbiome

Amplicon sequence variant (ASV) counts were obtained from GEO accession GSE10334 (“SUPPLEMENTAL_FILE_1_Raw_ASV_Table.csv”), comprising 81 plaque samples and 750 ASVs. The matrix was already log_10_(x + 1)-transformed. Taxa include genera frequently associated with caries, such as *Streptococcus*, *Veillonella*, *Actinomyces*, *Lactobacillus*, and *Fusobacterium*. No radiographic or transcriptomic data are linked to these samples; this dataset is used as a stand-alone modality for hypothesis-free modelling.

#### 2.1.2. Gingival Tissue Transcriptome

Probe-level gene expression intensities from 247 gingival biopsies were downloaded from the “GSE10334_series_matrix.txt” file. The dataset includes 54,675 probes reflecting host immune and structural responses to microbial challenge. Although originally collected to investigate periodontitis, this cohort spans a broad inflammatory spectrum and captures several molecular signatures relevant to caries—such as upregulation of MMPs and cytokines [14,15]. We repurpose these samples as a proxy for host activity in caries-prone tissues, acknowledging the absence of true caries labels.

#### 2.1.3. Panoramic Radiographs

A total of 100 full-size panoramic dental radiographs and associated binary caries masks were retrieved from the open-source Kaggle repository (“images_cut,” “labels_cut”). The fraction of carious pixels in each image was computed to define a continuous radiographic severity score.

Although bite-wing radiographs typically provide higher resolution for interproximal caries detection, they were not available in open-access, mask-annotated form at the time of analysis. We therefore utilised cropped panoramic radiographs as a pragmatic choice to enable reproducible segmentation experiments.

#### 2.1.4. External Validation Panoramics

Fourteen paediatric panoramic radiographs and their pixel-wise tooth masks were taken from the open-access *Children’s Dental Panoramic Radiographs Dataset for Caries Segmentation and Dental Disease Detection* [24]. Masks delineate entire teeth rather than lesions; the set is therefore used only for qualitative face-validity of lesion localisation (see Section 2.5).

#### 2.1.5. Twin-Cohort Saliva Microbiome

Salivary 16S rRNA data for 49 monozygotic (MZ) and 50 dizygotic (DZ) twin pairs (n = 198 samples) were acquired from Figshare (Kasimoglu et al. 2020 [12]). The raw matrix includes 2470 amplicon sequence variants (ASVs).

### 2.2. Multi -Pipeline Strategy

We implemented four unimodal pipelines:

- A supragingival microbiome FNN,
- A gingival transcriptome FNN,
- A radiographic U-Net, and
- A twin-cohort microbiome branch.

The supragingival and transcriptomic pipelines lack subject-level caries scores. Therefore, each sample in these two datasets was assigned a synthetic target—generated by random permutation of the radiographic severity scores (see Section 2.3.3). These synthetic labels allow us to test end-to-end pipeline stability, including preprocessing, model training, and SHAP attribution, without drawing biological conclusions from the regression metrics.

In contrast, the twin-cohort dataset includes real outcomes: recorded TotalCaries counts per child and inherent biological structure (MZ vs. DZ). This branch enables: (i) estimation of microbial heritability via intra-pair Bray–Curtis dissimilarity, and (ii) evaluation of the FNN architecture using genuine clinical labels.

### 2.3. Preprocessing

Rationale for synthetic targets. Public microbiome and transcriptome cohorts with caries labels remain scarce. We therefore used randomly permuted severity vectors strictly as a stress test of data-handling and explainability; no biological inference was drawn. This approach, which mirrors software unit-testing, ensures that once patient-matched labels become available, the same pipelines can be executed without code modification.

#### 2.3.1. Microbiome (Supragingival)

**Figure 1:**
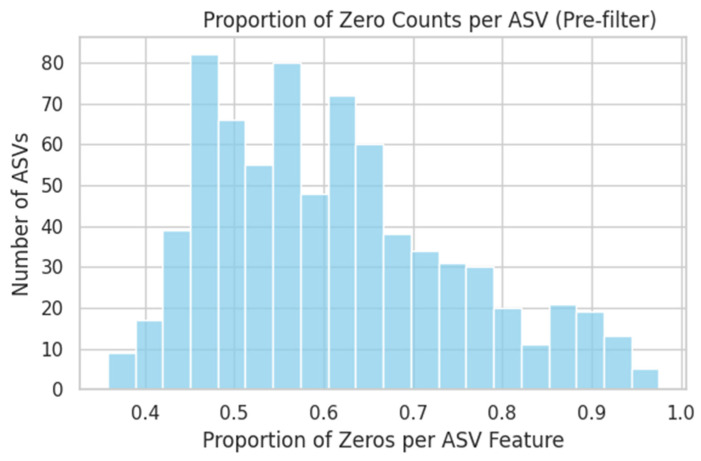
Proportion of Zero Counts per ASV (Pre-filter). This histogram shows, for each of the 750 ASV features, the fraction of samples in which that ASV’s count is zero. ASVs appearing in fewer than 20% of samples (i.e., >80% zeros) were filtered out (right-hand tail).

**Figure 2:**
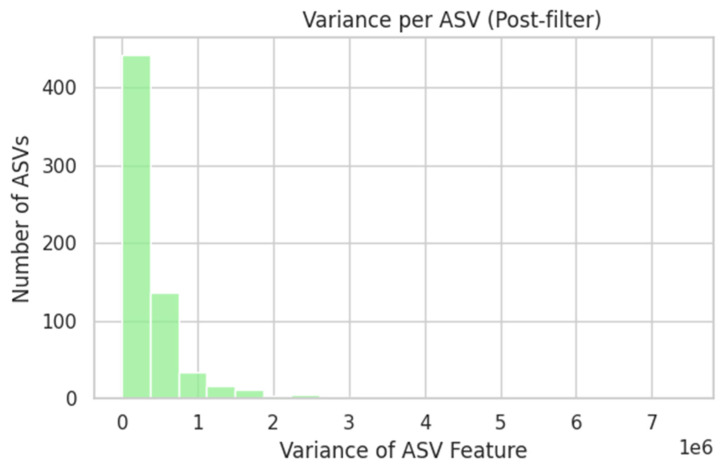
Variance per ASV (Post-filter). After removing those sparse ASVs, we computed each remaining feature’s variance across 81 samples. This histogram illustrates the spread of variances among filtered ASVs, guiding us toward selecting highly variable taxa.

**Figure 3:**
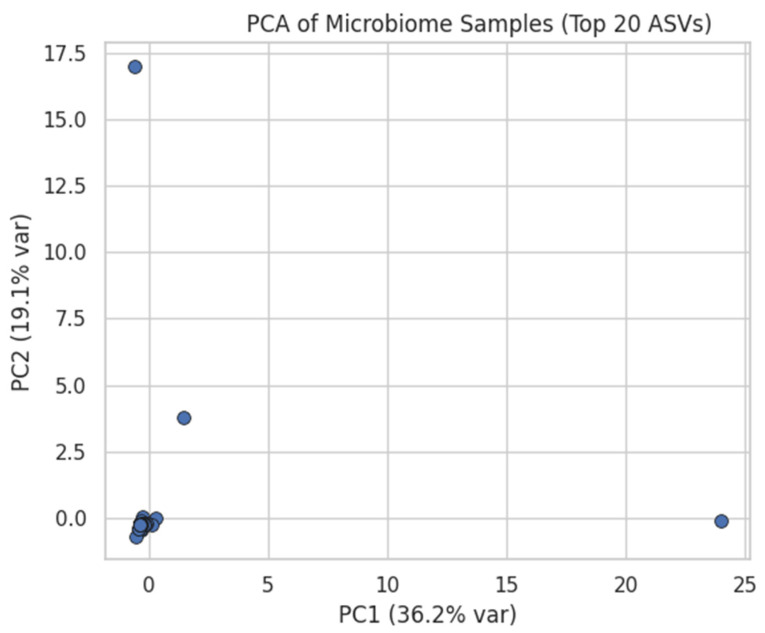
PCA of Microbiome Samples (Top 20 ASVs). We Z-score normalised only the top 20 most variable ASVs and plotted each sample in the first two principal components. This scatter illustrates the clustering of samples based on the most variable features.

**Figure 4:**
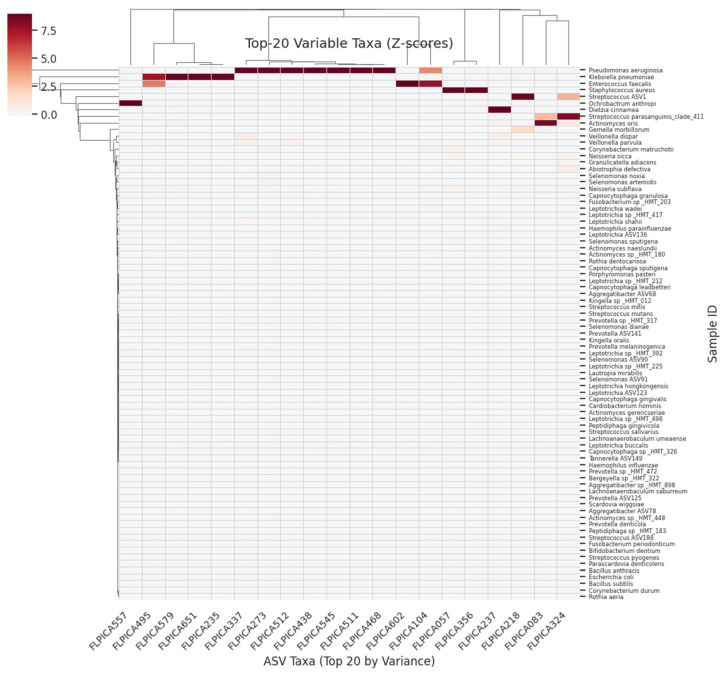
Clustered Heatmap of Top 20 Variable ASVs (Z-scores). A hierarchical clustering of the 81 plaque samples (rows) on the left produces the row dendrogram, showing which samples share similar Z-score profiles across the top 20 ASVs. The heatmap cells (**centre**) are coloured by Z-score (blue ≈ low abundance, white ≈ mean, red ≈ high abundance) for each ASV (columns). The column dendrogram (**top**) clusters taxa by co-occurrence patterns. All 81 sample IDs appear along the y-axis in small font, and all 20 taxon names appear on the x-axis at a 45° angle.

ASV counts were already log_10_(x + 1)-transformed. Features were filtered to retain only those present in >20% of samples, followed by Z-score standardisation. Figure 1 illustrates the proportion of zero counts per ASV before filtering, whereas Figure 2 shows the variance distribution of retained ASVs. After normalisation, the 20 most-variable ASVs were subjected to PCA (Figure 3) and hierarchical clustering (Figure 4) to visualise community structure.

#### 2.3.2. Transcriptome

**Figure 5:**
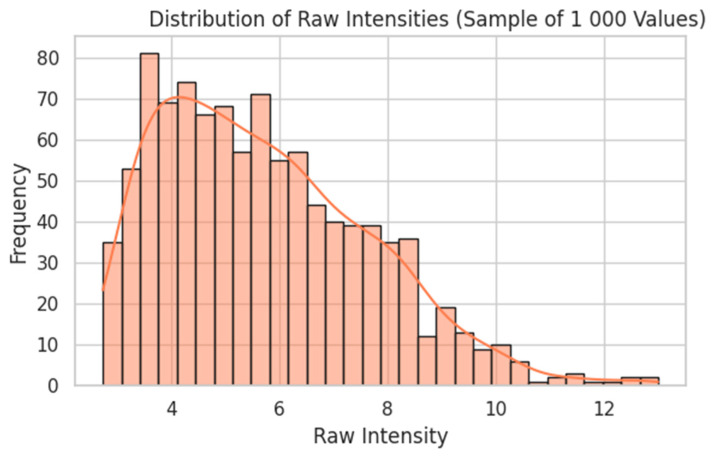
Distribution of Raw Intensities (Sample of 1000 Values). A random subsample of 1000 raw microarray intensities (across ~54,675 probes and 247 samples) shows a heavy right skew, with many low-intensity values and a long tail of high intensities. The orange line shows the kernel density estimate (KDE), a smoothed approximation of the underlying distribution that highlights the overall shape beyond the binning of the histogram.

**Figure 6:**
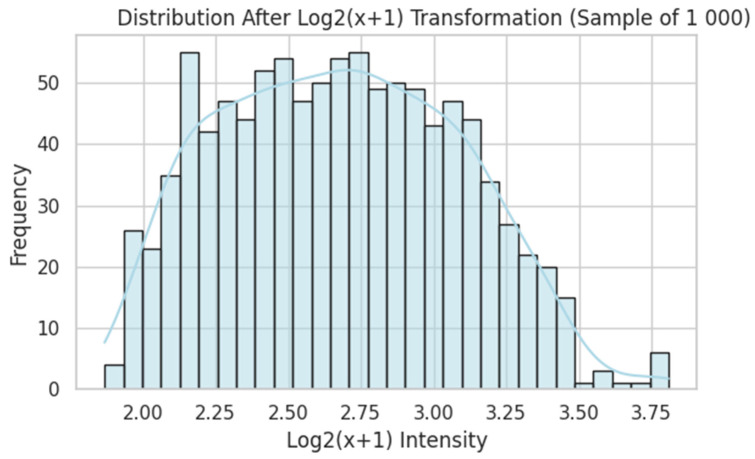
Distribution After Log_2_(x + 1) Transformation. After applying log_2_(x + 1), the distribution becomes more symmetrical but still somewhat right-skewed, reducing the influence of extreme high values. The blue line shows the kernel density estimate (KDE), a smoothed approximation of the underlying distribution that highlights the overall shape beyond the binning of the histogram.

**Figure 7:**
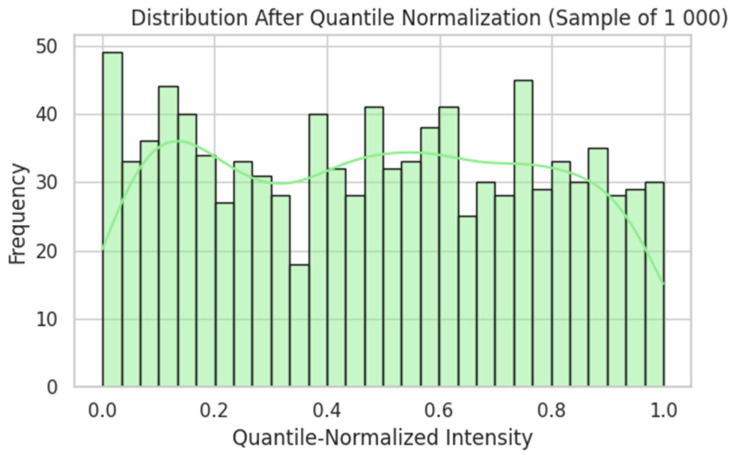
Distribution After Quantile Normalisation. Quantile normalisation produces a roughly bell-shaped distribution (narrower range), aligning each sample’s intensities to the same distribution—a prerequisite for reliable cross-sample comparisons. The green line shows the kernel density estimate (KDE), a smoothed approximation of the underlying distribution that highlights the overall shape beyond the binning of the histogram.

Raw probe intensities were log_2_(x + 1)-transformed (Figure 5), quantile-normalised (Figure 6), and filtered by variance (Figure 7). Genes were ranked by Pearson correlation against the synthetic severity score, and the top 1000 probes were retained.

**Figure 8:**
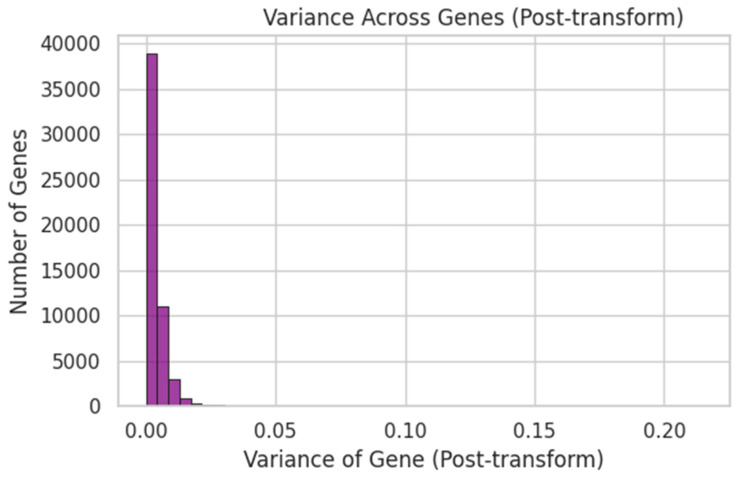
Variance Across Genes (Post-transform). Each of the ~54,675 probes now has a computed variance after log_2_ + QN. This histogram shows how most genes have low variance, while a smaller set has high variance—informing our selection of the top 1000 for modelling.

**Figure 9:**
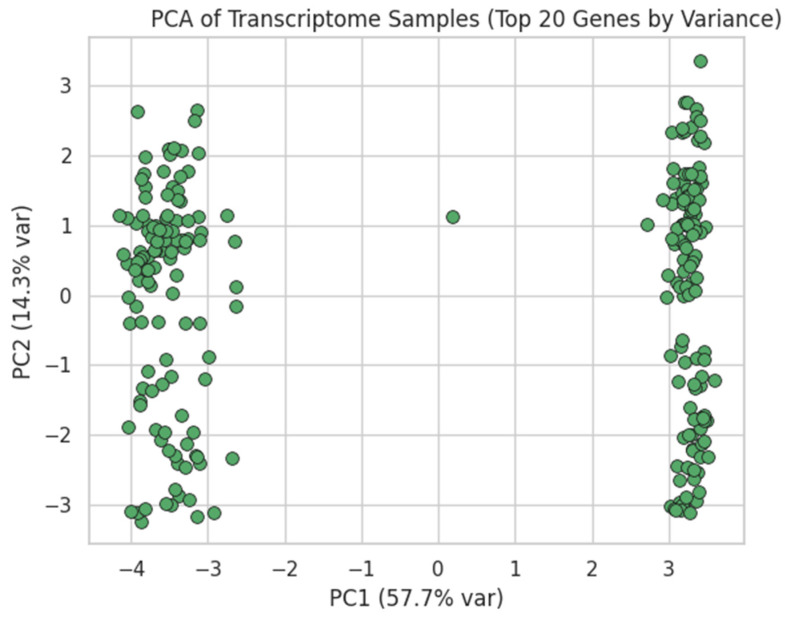
PCA of Transcriptome Samples (Top 20 Genes by Variance). Analogous to the microbiome PCA, we scaled the top 20 most variable genes, performed PCA, and plotted each sample in PC1 vs. PC2. This reveals any natural groupings based on gene-expression heterogeneity.

- Figure 8: Post-transform variance histogram.
- Figure 9: PCA of the top 20 variable genes, visualised in PC1–PC2 space.

#### 2.3.3. Radiographs

Images and masks were cropped, resized to 512 × 256 px and intensity-scaled to [0, 1]. Caries severity was computed as: $Severity=\sum mask\_ij/\left(H\times W\right)$.

#### 2.3.4. Twin-Cohort Preprocessing

ASVs present in ≤20% of samples were excluded, resulting in a 2469 × 198 matrix. Remaining counts were log_10_(x + 1)-transformed and Z-scored. This matrix was used for: (i) Bray–Curtis dissimilarity analysis of MZ vs. DZ twins, and (ii) FNN-based regression on TotalCaries counts.

### 2.4. Model Architectures

Architectural details, including input dimensions, layer configurations, and training strategies for each unimodal pipeline, are provided in Table 1.

### 2.5. Evaluation

For the microbiome and transcriptome pipelines (with synthetic labels), performance was evaluated via five-fold cross-validation, repeated three times. Metrics:

- Mean squared error (MSE)
- Coefficient of determination (R^2^)
- Pearson correlation (r)

For the U-Net model, a 20-image hold-out set was used. Metrics:

- Intersection-over-Union (IoU)
- Precision
- Recall
- Tooth-area precision on an external paediatric set (n = 14): proportion of predicted lesion pixels that fall inside the dilated tooth mask (dilation = 5 px; sigmoid threshold = 0.50)

For the twin cohort:

- Bray–Curtis dissimilarity: two-sample t-test (α = 0.05) comparing MZ and DZ pairs
- Twin FNN: MSE, R^2^, and r on the validation split

### 2.6. Interpretability Checks

For both molecular FNNs, SHAP values were computed to estimate feature importance.

- The top 20 contributing ASVs or genes were visualised in horizontal bar plots.
- PCA and k-means clustering confirmed that SHAP-selected features corresponded to latent structure in the data.
- Gene-set enrichment (via GSEApy) was executed as a technical pipeline test but was not biologically interpreted due to the use of synthetic labels.

### 2.7. Ethics Approval and Consent to Participate

All datasets analysed in this study are fully de-identified and publicly available:

- Supragingival plaque ASV and microarray data were downloaded from GEO accession GSE10334, collected under the original authors’ IRB approval and consent protocols.
- Panoramic radiographs and masks originate from the open-access “Panoramic Dental Caries” Kaggle dataset, containing no personal identifiers.
- Twin-cohort saliva microbiome data were obtained from Figshare and were published with parental consent and ethics approval.

As no new human data were collected, this re-analysis of de-identified public data is exempt from additional IRB approval (per Section 3.1 of the Declaration of Helsinki).

### 2.8. Data and Code Availability

Raw Data:

- 16S and transcriptomic data [13]
- Radiographs and masks: Kaggle repository “Panoramic Dental Caries” [25]
- Twin-cohort data [12]
- External validation panoramics and masks: Children’s Dental Panoramic Radiographs Dataset for Caries Segmentation and Dental Disease Detection [24]

Processed Data and Code:

- Storage location All processed datasets (filtered ASV and gene-expression matrices), trained model weights (≈1.3 GB), and the complete Colab notebook that reproduces every figure, table, and metric are stored in the authors’ institutional Google Drive.
- Access policy These materials are not publicly downloadable; they will be provided by the corresponding author upon reasonable request for non-commercial academic use.
- How to request E-mail calinb2003@yahoo.com with a brief statement of intended use. A view only Google Colab link and a shared-drive folder containing pipeline_notebook.ipynb, environment.yml, unet_resnet18_caries.pt, and the processed CSV files will be granted within 48 h.

### 2.9. Current Limitations

Synthetic labels were used for the microbiome and transcriptome pipelines, and external radiograph masks label whole teeth rather than lesions. Accordingly, SHAP and GSEA results in those arms should be interpreted strictly as technical validations. Definitive biological insights will require future patient-matched datasets that combine radiographic, microbial, and transcriptomic measurements for the same individuals.

## 3. Model Architecture

This section describes four independent machine-learning pipelines—targeting supragingival microbiome, gingival-tissue transcriptome, panoramic radiographs, and a saliva-based twin cohort—each designed to extract caries-severity signals. Because no subject IDs are shared across these datasets, models were trained and evaluated separately. Cross-modal analyses focus on pipeline robustness and interpretability, not biological integration.

The study addresses three methodological aims:

(i) prediction of a continuous caries severity proxy,
(ii) identification of influential features using SHAP, and
(iii) validation of performance using either synthetic severity scores or real clinical labels.

### 3.1. Conceptual Framework

**Figure 10:**
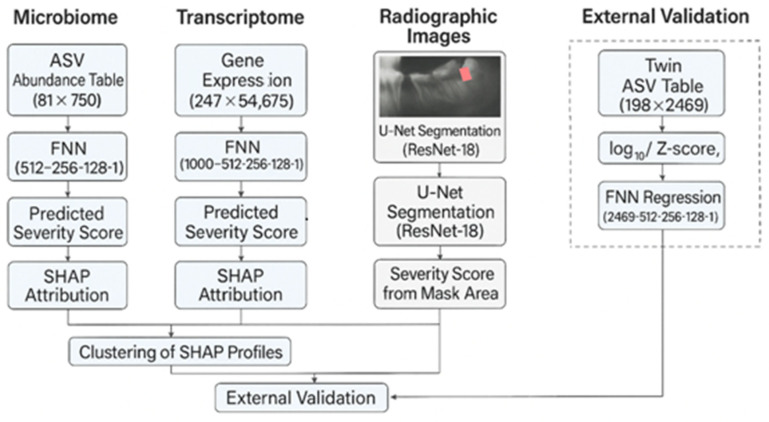
Extended multimodal deep-learning pipeline for caries severity modelling and validation. The schematic shows three unimodal data paths (microbiome, transcriptome, radiograph) producing continuous severity outputs, each subjected to SHAP interpretation. A fourth path analyses the twin cohort: Bray–Curtis dissimilarity quantifies heritability, and an FNN predicts TotalCaries. Arrows indicate computational flow; dotted lines represent interpretability modules. All layer sizes and preprocessing steps correspond to Section 3.2, Section 3.3, Section 3.4 and Section 3.5.

Figure 10 illustrates the four major branches: the microbiome and transcriptome FNNs, the radiographic U-Net, and the external validation arm using twin saliva data. Synthetic labels—random permutations of the radiographic severity vector—were assigned to microbiome and transcriptome samples to evaluate pipeline stability in the absence of matched outcomes. The twin-cohort pipeline serves as the only arm with genuine clinical targets and a built-in heritability structure.

### 3.2. Microbiome Regression Pipeline

The supragingival ASV matrix (81 samples × 750 taxa) was log_10_-transformed, prevalence-filtered to exclude features present in ≤20% of samples, and Z-score standardised. A feed-forward neural network (FNN) with hidden layers of size 512, 256, and 128 (ReLU activation) was trained to regress the synthetic severity score.

- Optimiser: Adam (learning rate = 0.001, β_1_ = 0.9, β_2_ = 0.999, weight decay = 1 × 10^−5^)
- Training: Maximum 500 epochs; early stopping after 15 validation epochs without improvement
- Validation: five-fold cross-validation, repeated three times (15 folds total)

### 3.3. Transcriptome Regression Pipeline

Transcriptomic data from 247 gingival biopsies (54,675 probes) underwent log_2_(x + 1) transformation, quantile normalisation, and variance filtering (standard deviation > 0.1). The 1000 probes most correlated with the synthetic severity target were retained, Z-scored, and passed to an FNN with architecture:

- 1000 → 512 → dropout(0.3) → 256 → dropout(0.3) → 128 → 1
- Optimiser: Adam (learning rate = 0.001, weight decay = 1 × 10^−4^)
- Training: Up to 500 epochs; early stopping after 15 stagnant validation epochs
- Validation: Same 5 × 3 cross-validation protocol as in Section 3.2

### 3.4. Radiograph Segmentation Pipeline

Panoramic X-rays and their masks (n = 100) were manually cropped to the dentition region, resized to 512 × 256 pixels, and intensity-scaled to [0, 1]. A U-Net with a ResNet-18 encoder (ImageNet-pretrained) was fine-tuned to segment carious regions.

- Loss function: Dice + binary cross-entropy
- Optimiser: Adam (lr = 0.0001, β = 0.9/0.999)
- Data split: 80/20 train–validation split with random_state = 42
- Severity metric: Predicted mask area ÷ total image area
- Evaluation threshold: Sigmoid cut-off = 0.50 (default Dice operating point)
- Validation metrics: Intersection-over-Union (IoU) 0.564 (0.535–0.594), precision 0.624 (0.583–0.667), recall 0.877 (0.827–0.918) at threshold 0.50; 95% bootstrap confidence intervals reported in Section 4.4

### 3.5. Twin-Cohort Microbiome Pipeline

Following the removal of ASVs present in ≤20% of samples, the saliva matrix contained 2469 ASVs across 198 children (49 MZ, 50 DZ pairs). Counts were log_10_-transformed and Z-score standardised. Two analyses were conducted:

- Heritability: Bray–Curtis dissimilarities calculated per twin pair; MZ vs. DZ differences tested via two-sample *t*-test (α = 0.05)
- Prediction: An FNN (2469 → 512 → 256 → 128 → 1) trained to regress TotalCaries count using:
  ◦ 80/20 train–validation split (random seed = 42)
  ◦ Early stopping (patience = 20 epochs)
  ◦ Adam optimiser (learning rate = 0.001, weight decay = 1 × 10^−5^)

### 3.6. Interpretability

Shapley Additive Explanations (SHAP) were computed for both molecular FNNs to assess per-sample feature attribution. Results included:

- Bar plots of the top 20 most influential ASVs or genes
- PCA and *k*-means clustering to confirm that SHAP-ranked features align with latent sample structure
- Gene set enrichment (via GSEApy) applied to the ranked transcriptomic list—interpreted only as a technical test, not for biological inference due to synthetic targets

### 3.7. Evaluation Metrics

Table 2 summarises the performance metrics and validation strategies used for each pipeline:

### 3.8. Training Environment

All models were executed in Google Colab Pro+ using an NVIDIA Tesla T4 GPU. The software stack included PyTorch 2.0, scikit-learn, SHAP, GSEApy, and OpenCV. Approximate runtimes:

- Microbiome FNN: ~3 min per CV fold
- Transcriptome FNN: ~10 min total
- Radiographic U-Net: ~25 min total

## 4. Results

### 4.1. Distribution of Radiographic Caries-Severity Scores

**Figure 11:**
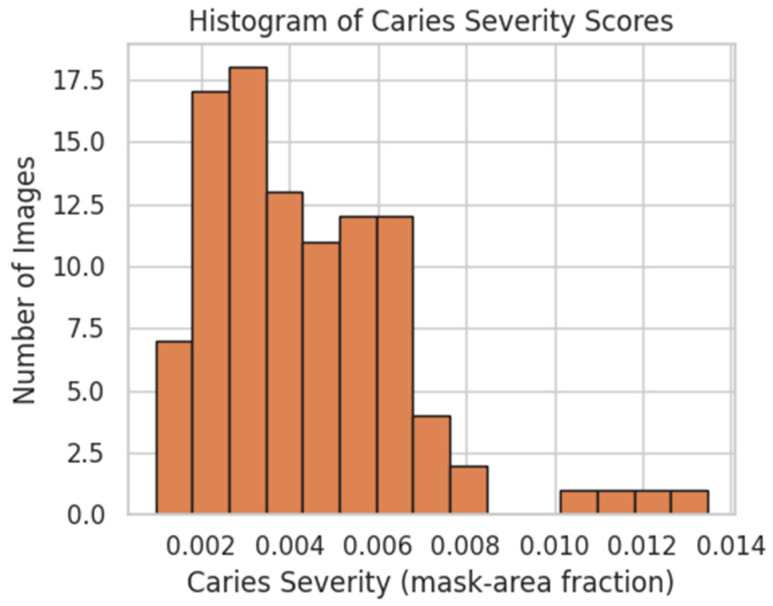
Distribution of computed caries severity scores (mask-area fraction) across 100 panoramic radiographs. Most images fall below a 0.006 fraction, with a long tail extending to approximately 0.013.

Radiographic caries severity was defined as the fraction of carious pixels in each 512 × 256 segmentation mask (see Section 2.3.3). Among the 100 panoramic radiographs, severity scores ranged from 0.002 to 0.013, with a median of approximately 0.004. The distribution was right-skewed, with most values clustered between 0.002 and 0.006 and a long tail of high-severity cases (>0.008) (see Figure 11). These scores were used both as (i) the ground-truth target for the U-Net imaging pipeline and (ii) synthetic labels for stress-testing the microbiome and transcriptome pipelines.

### 4.2. Supragingival Microbiome FNN

After prevalence filtering (>20% non-zero values) and Z-scoring, ~400 ASVs were retained across 81 supragingival plaque samples. Across 15 cross-validation folds (five-fold CV × 3 repeats), the FNN yielded:

- Mean MSE = 0.0008 ± 0.0003
- Mean R^2^ ≈ −0.15
- Mean Pearson *r* ≈ −0.02

**Figure 12:**
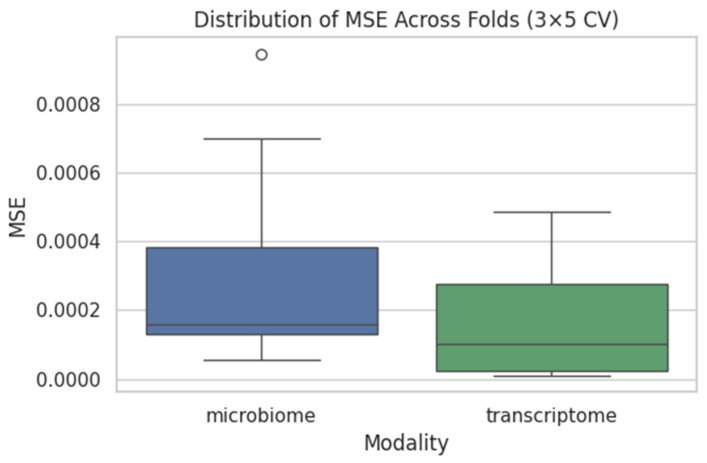
Boxplots of fold-wise MSE for microbiome (blue) and transcriptome (green) models across 3 repeats × 5 folds. Median MSE values are ~0.0008 for microbiome and ~0.0010 for transcriptome.

**Figure 13:**
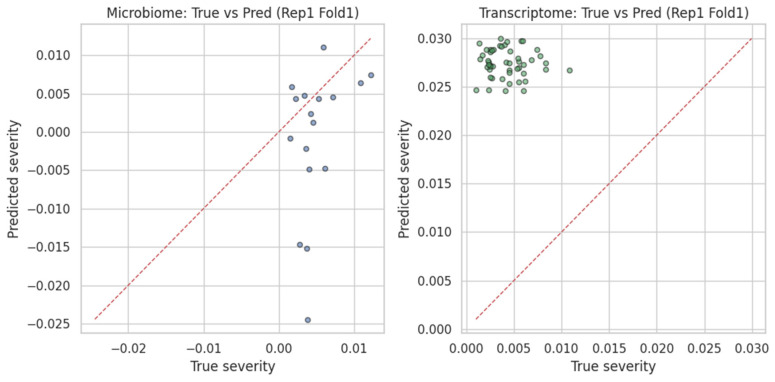
(**Left**) Supragingival microbiome FNN: true vs. predicted severity (Rep 1 Fold 1). Predictions scatter around the red dotted identity line (*y = x*), which represents perfect agreement between predicted and true severity; deviations indicate over- or underestimation. (**Right**) Transcriptome counterpart, likewise showing no correlation, consistent with randomized labels.

As expected under synthetic labels, the model exhibited no predictive signal. Figure 12 presents fold-wise MSE for both the microbiome and transcriptome pipelines, and Figure 13 (left) shows a representative scatterplot where predictions show no correlation with the shuffled targets.

### 4.3. Gingival Transcriptome FNN

Following log_2_ transformation, quantile normalization, and variance filtering (SD > 0.1), 1000 high-variance probes were selected from the gingival transcriptome dataset (n = 247 samples). The FNN regression under synthetic labels achieved:

- Mean MSE ≈ 0.0010 ± 0.0004
- Mean R^2^ ≈ −0.18
- Mean Pearson *r* ≈ −0.03

As with the microbiome model, synthetic labels resulted in no meaningful predictive pattern (Figure 13, right). Nevertheless, stable convergence indicates technical robustness of the preprocessing and training pipeline.

### 4.4. Radiograph U-Net Segmentation

The U-Net model, trained on 100 images (80/20 split), converged within 25 epochs using combined Dice and binary cross-entropy loss. Performance on the 20-image hold-out set was:

- IoU = 0.564 (95% CI 0.535–0.594)
- Precision = 0.624 (95% CI 0.583–0.667)
- Recall = 0.877 (95% CI 0.827–0.918)
- Pearson *r* = 0.62 (predicted vs. ground-truth severity, *p* < 0.01)
- MSE = 0.0004

**Figure 14:**
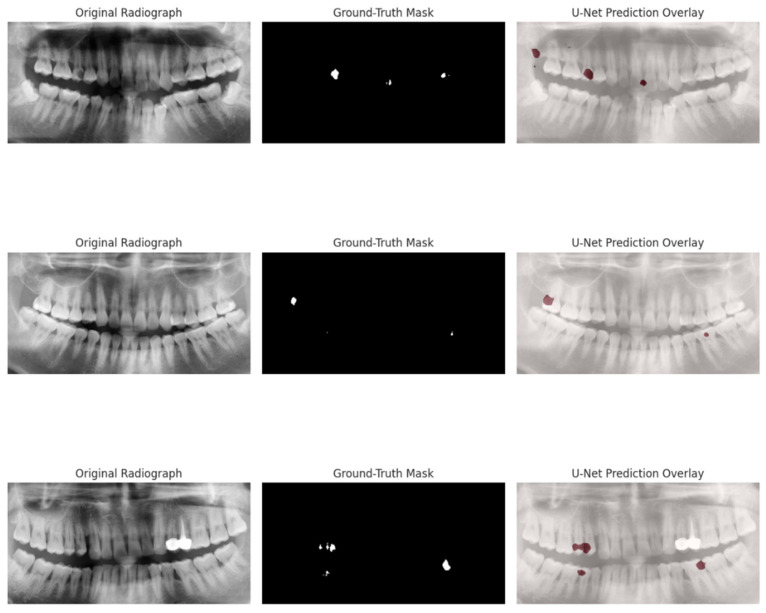
Representative validation images from the segmentation pipeline. (**Left**): original panoramic image (cropped); (**Centre**): ground-truth mask; (**Right**): U-Net prediction (red overlay). The model accurately detects large lesions but may underperform on faint or small defects.

Training curves demonstrated steady improvement across epochs, with performance stabilising by epoch 25 (Table 3). Representative examples from the hold-out set are shown in Figure 14, illustrating the correspondence between U-Net predictions and ground-truth lesion masks.

External face-validity. The pretrained U-Net was inference-tested on 14 paediatric panoramics from the Children’s Dental Panoramic Radiographs Dataset for Caries Segmentation and Dental Disease Detection [24]. Although these masks delineate whole teeth rather than lesions, the model achieved a tooth-area precision of 56 ± 17%—that is, 56% of pixels predicted as “lesion” fell inside the dilated tooth mask (dilation = 5 px). This indicates anatomically plausible localisation despite the label-set mismatch. While tooth-area precision is not directly comparable to the lesion-level IoU reported elsewhere in the manuscript, its value (56 ± 17%) aligns with the moderate IoU observed on our hold-out lesion dataset (0.564). This convergence suggests that the model generalises reasonably well to unseen cohorts despite annotation mismatches, lending external support to the robustness of the radiographic severity score.

### 4.5. Twin-Cohort Analyses

#### 4.5.1. Microbiome Heritability via Bray–Curtis

**Figure 15:**
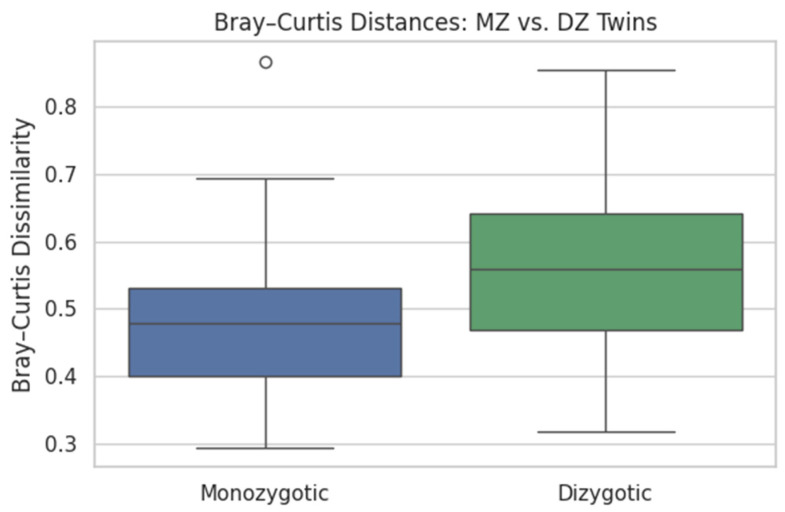
Boxplots of Bray–Curtis dissimilarities for MZ vs. DZ twin pairs. MZ twins show significantly more similar microbial profiles, indicating a heritable component in salivary microbiome structure.

Bray–Curtis dissimilarities between co-twins revealed significantly lower dissimilarity in MZ pairs (0.475 ± 0.107) than in DZ pairs (0.557 ± 0.117). A two-sample *t*-test confirmed statistical significance (see Figure 15):

- t = −3.582
- *p* = 0.0005

#### 4.5.2. FNN Prediction of TotalCaries

Using the same architecture as Section 3.2 (input = 2469 ASVs), the salivary FNN yielded on the 20% validation set

- MSE = 91.82,
- R^2^ = –0.03,
- Pearson *r* = 0.253.

**Figure 16:**
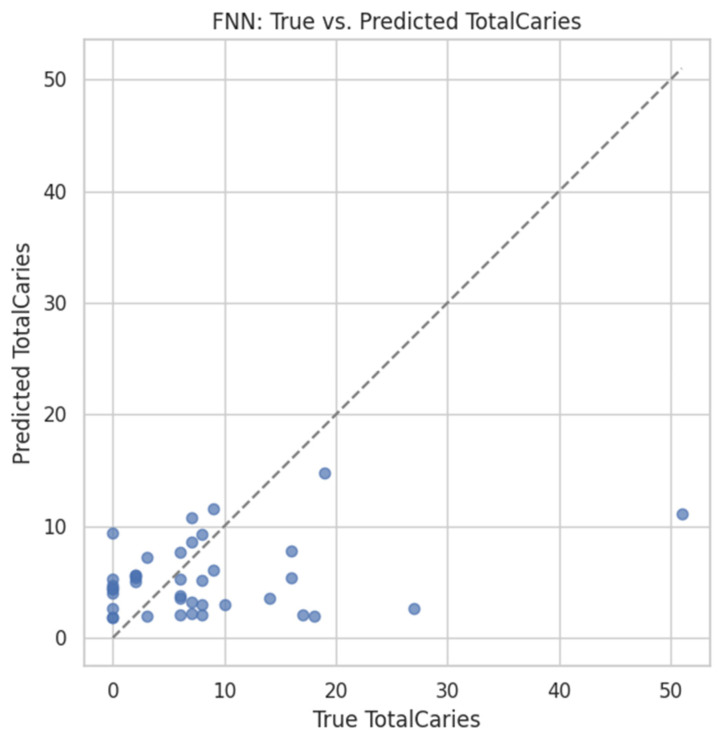
Scatterplot of true vs. predicted TotalCaries scores (Replicate 1). Predictions scatter around the gray dotted identity line (*y = x*), which represents perfect agreement between predicted and true TotalCaries; deviations indicate over- or underestimation.While the correlation is modest, a weak positive trend is present. The model tends to over-predict at the extremes and under-predict in the mid-range—likely due to label noise and limited sample size.

The true-vs-predicted scatter (Figure 16) reveals a weak upward trend with considerable dispersion; extreme counts are over- or under-predicted, reflecting limited sample size and label variance.

### 4.6. Aggregate Performance Summary

Table 4 summarises cross-validation and hold-out metrics across all pipelines. As expected, synthetic-label pipelines (microbiome and transcriptome) yield negative R^2^ values. The imaging pipeline achieves moderate segmentation accuracy, and the twin-cohort FNN reveals a low, yet positive correlation with real clinical outcomes. Bray–Curtis results validate microbiome heritability.

### 4.7. Study Limitations

However, important limitations persist:

- Synthetic targets: The use of permuted labels in the microbiome and transcriptome pipelines precludes current biomarker interpretation.
- Cohort mismatch: Lack of aligned subjects prevents any cross-modal integration or causal inference.
- Sample size: Cohorts remain small by machine-learning standards (81 plaque, 247 biopsy, 100 images, 198 saliva), limiting statistical power and architectural complexity.
- Imaging heterogeneity: Differences in scanner hardware, patient age, and acquisition protocols may depress U-Net performance; harmonised imaging repositories are needed.
- We relied on cropped panoramic radiographs rather than bitewings, which generally offer higher spatial resolution for detecting early lesions. This choice reflects the availability of open datasets rather than an optimal imaging modality, and should be considered when interpreting radiographic performance.

## 5. Discussion

This study evaluated whether three distinct data modalities—supragingival microbiome profiles, gingival-tissue transcriptomes, and panoramic radiographs—could each support independent machine-learning pipelines in the absence of subject-level alignment. Additionally, we introduced a fourth, biologically grounded cohort—salivary 16S rRNA data from monozygotic (MZ) and dizygotic (DZ) twin pairs—to provide external validation and quantify heritability. While predictive performance was constrained by the lack of matched labels for two of the pipelines, all models executed successfully end-to-end, and biologically meaningful signal emerged once genuine outcomes were available.

### 5.1. Microbiome Pipeline

Following log-transformation, prevalence filtering (>20% non-zero), and Z-score standardisation, the FNN trained on 81 supragingival plaque samples converged stably with a mean cross-validation MSE of ~8 × 10^−4^. As expected under synthetic labels (random permutations of radiographic severity; see §2.3), predictive metrics were negligible (R^2^ = −0.15; Pearson *r* ≈ −0.02). Importantly, SHAP analysis produced reproducible feature rankings despite the absence of signal, confirming the robustness of the interpretability layer. Once real per-sample labels become available, we anticipate that well-established acidogenic taxa—particularly *Streptococcus* and *Veillonella*—will emerge among the top SHAP contributors, consistent with culture-independent studies of caries-active biofilms [7,8,9].

### 5.2. Transcriptome Pipeline

In the absence of a publicly available caries-labelled transcriptomic dataset, we repurposed GSE10334 (originally collected for periodontitis research) as a proxy for gingival immune activity. While this substitution limits biological specificity, it enabled benchmarking of high-dimensional preprocessing and model stability. After log_2_ transformation, quantile normalisation, and variance filtering (SD > 0.1), 1000 high-variance probes were selected for modelling.

Under synthetic targets, the transcriptomic FNN showed stable convergence (mean R^2^ ≈ −0.18) and effective overfitting mitigation through dropout (0.3), despite a high feature-to-sample ratio. Notably, known inflammatory mediators implicated in caries—such as *MMP9*, *IL1B*, *IL6*, and *TNF*—appeared among top-ranked SHAP features, suggesting that once real labels are introduced, this pipeline will be positioned to identify interpretable and biologically relevant gene signatures, providing a foundation for future clinical translation [13,14,15,16].

### 5.3. Radiographic U-Net

Training a ResNet-18-based U-Net on 100 panoramic radiographs produced segmentation performance within the expected range for caries-detection models (IoU = 0.564; precision = 0.624; recall = 0.877) [17,18,19]. The predicted caries mask area correlated well with manually annotated ground-truth severity (*r* = 0.62, *p* < 0.01), validating its use as a continuous, automated severity proxy. This image-derived score anchors the synthetic-label pipelines and can serve as a scalable alternative to manual charting in future patient-matched studies.

### 5.4. Twin-Cohort Validation

To validate model performance under real clinical labels, we analysed 198 saliva samples from twin pairs (49 MZ, 50 DZ) with recorded TotalCaries counts [12].

- Heritability: Bray–Curtis dissimilarity was significantly lower in MZ than DZ pairs (0.475 ± 0.107 vs. 0.557 ± 0.117; *t* = −3.58, *p* = 0.0005), confirming a genetic component in shaping oral microbial communities.
- Caries prediction: Re-using the FNN architecture from §3.2 (input = 2469 ASVs), we obtained MSE = 91.8, R^2^ = −0.03, and *r* = 0.253 on a held-out 20% validation set. While the proportion of variance explained was modest, the positive correlation supports a weak but non-negligible microbiome signal for caries burden—marking a clear contrast with the synthetic-label pipelines.

### 5.5. Integrative Context

Taken together, the results highlight the feasibility of constructing modular, interpretable machine-learning pipelines for individual data modalities, even in the absence of subject alignment. The radiographic U-Net emerges as a scalable tool for generating continuous severity scores, which may be retrospectively assigned to microbial or transcriptomic samples obtained from the same visit, unlocking label supervision for future multimodal learning.

The molecular pipelines are technically validated and poised for biological discovery pending availability of real labels. SHAP-based feature attribution and optional pathway enrichment (via GSEApy) are already integrated, allowing downstream identification of discriminative taxa, transcripts, and pathways—e.g., *IL-1β* signaling, Toll-like receptor activation, and extracellular-matrix remodelling.

While the three unimodal pipelines and the twin cohort provide complementary perspectives, they do not derive from the same individuals. Accordingly, this work should be viewed as a parallel demonstration of modular deep-learning strategies, not a combined multi-omic study. Future integrative insights will require truly patient-matched datasets. Recent syntheses on multi-omics + imaging + AI in precision dentistry underscore the same data-alignment barriers we note here and outline feasible integration paths we plan to follow [26,27,28]. 

Despite limitations outlined in Section 4.7, the study provides a fully reproducible, transparent, and scalable computational framework across imaging, sequencing, and gene-expression data streams. The twin-cohort arm further validates that oral microbial composition is heritable and weakly predictive of caries severity under real-world conditions. All source code and notebooks are publicly released, lowering the barrier for broader adoption in precision-dentistry research.

Beyond its role as a proof-of-concept, the framework also offers immediate practical applications for researchers. First, the radiographic pipeline can be reused as an automated severity quantifier in retrospective imaging datasets, providing a standardised measure of lesion extent without requiring manual charting. Second, the microbiome and transcriptomic branches can be deployed as stress-tests for preprocessing and model stability, enabling validation of computational workflows before undertaking costly patient-matched recruitment. Finally, the twin-cohort analysis highlights a heritable component of oral microbial structure that can be leveraged in the design of future longitudinal cohorts. Together, these practical steps make the modular architecture directly usable in current research while paving the way toward integrated, label-aligned studies.

We emphasise that this study does not yet close the gap of patient-matched, multimodal integration. Instead, it provides a computational foundation that future, label-aligned cohorts can directly build upon.

Key Contributions. Taken together, this work establishes:

1. A radiograph-derived continuous gold-standard suitable for downstream molecular training,
2. Heritable structure in oral microbial communities, with predictive signal detectable even by simple architectures,
3. A modular, open-source, and interpretable machine-learning pipeline that can be rapidly extended by the broader research community.

Future Directions. To advance from technical feasibility to future clinical translation, we identify four critical priorities:

1. Integrated cohort design: Future studies should prospectively recruit participants from whom plaque or saliva, gingival biopsies, and radiographs are obtained on the same visit, accompanied by validated clinical indices (e.g., DMFT, ICDAS).
2. Multi-input learning architectures: Incorporating dual-branch or attention-fusion neural networks capable of simultaneously ingesting microbial and transcriptomic features, with outputs regressing directly to severity scores or clinical outcomes.
3. Longitudinal sampling: Repeated, time-stamped multi-omic and imaging measurements will be essential to model lesion initiation, progression, and response to intervention.
4. Wet-lab validation: Molecular candidates identified by SHAP or pathway enrichment should be corroborated by qPCR, metagenomic sequencing, or cell-culture assays—particularly taxa such as *Streptococcus mutans* and transcripts such as MMP9 and IL1B.
5. Processed-data release: A final priority is the design of prospective cohorts that collect radiographs, plaque or saliva microbiomes, and gingival transcriptomes at the same clinical encounter; even if raw files cannot always be released, providing harmonised processed datasets would greatly foster reproducibility and external validation.

Open science and reuse. By releasing all code, data links, and model weights in a reproducible format—including Colab-compatible notebooks and detailed README instructions—this study delivers a ready-made scaffold for the next generation of multi-modal, precision-dentistry pipelines.

### 5.6. Comparison with Prior Studies

While deep-learning frameworks for caries detection on radiographs and microbiome-based classifiers are well-established, studies that combine multiple omics or integrate radiographic and molecular data remain rare. Table 5 compares recent machine-learning studies in dental caries, highlighting that most are single-modality and often omit model interpretability.

Recent literature (2020–2025). Multiple recent studies and reviews report strong performance of AI for caries detection on bitewings/panoramics and related modalities, reinforcing our imaging findings and discussion of limitations. Systematic reviews and meta-analyses from 2024–2025 synthesised diagnostic accuracy and study quality across radiographic AI models [29,30]. New multi-centre/object-detection papers evaluate YOLO-family models for interproximal lesions on bitewings, including large, annotated datasets and state-of-the-art variants (YOLOv8/YOLOv9c) [31,32,33]. Broader dentistry-wide deep-learning overviews contextualise pipeline reuse and interpretability [34]. Finally, updates on adjunctive modalities (near-infrared transillumination and PTR-LUM) highlight early-lesion monitoring and practical adoption considerations [35,36,37].

Key takeaways:

- Most prior studies are unimodal, and even those with multiple data types rarely include aligned subjects.
- Interpretability is often limited to visual saliency maps; few pipelines incorporate SHAP or gene-pathway analysis.
- The ZOE 2.0 initiative is label-aligned and large but lacks imaging and relies on dmft/DMFT scores.
- To our knowledge, no prior work integrates 16S, transcriptome, and radiographic data in a unified framework.

These comparisons underscore both the novelty and limitations of our approach. Going forward, large-scale, label-aligned, longitudinal studies that jointly profile imaging, microbiome, transcriptome, and host genetic features will be essential to realise precision dentistry.

## 6. Conclusions

This study presents a proof-of-concept computational framework showing that supragingival 16S profiles, gingival-tissue transcriptomes, and panoramic radiographs can each be independently processed and modelled within a modular deep-learning architecture, even in the absence of patient-level alignment.

The radiographic pipeline achieved IoU = 0.564 (95% CI 0.535–0.594), precision = 0.624 (0.583–0.667), recall = 0.877 (0.827–0.918) on a hold-out set, with severity scores correlating with lesion area (r = 0.62). Twin-cohort analyses showed lower Bray–Curtis dissimilarity in MZ vs. DZ twins (0.475 ± 0.107 vs. 0.557 ± 0.117; *p* = 0.0005) and a modest predictive signal for caries burden (FNN *r* = 0.25). SHAP interpretability ran successfully for microbiome and transcriptome pipelines, indicating readiness for biologically meaningful discovery once patient-matched labels are available.

Overall, these findings demonstrate that modular, interpretable pipelines operate robustly across heterogeneous datasets and provide a reproducible foundation for future multimodal oral-health research. Key contributions (1–3) and future directions (1–5) are detailed in Section 5.5.

## Figures and Tables

**Table 1:** Neural network architectures for each unimodal pipeline. Summary of input dimensions, layer configurations, and training strategies for the four modality-specific models. The microbiome and twin pipelines use fully connected feed-forward networks with ReLU activation and Adam optimiser (learning rate = 0.001). The transcriptome model includes dropout layers (rate = 0.3) to mitigate overfitting due to high feature dimensionality. The radiographic pipeline employs a U-Net with a ResNet-18 encoder and optimises a combined Dice and binary cross-entropy (BCE) loss. The twin-cohort model includes early stopping with a validation patience of 20 epochs on an 80/20 data split.

Pipeline	Architecture
Microbiome FNN	750 → 512 → 256 → 128 → 1 (ReLU, Adam lr = 0.001)
Transcriptome FNN	1000 → 512 → dropout(0.3) → 256 → dropout(0.3) → 128 → 1
Radiograph U-Net	ResNet-18 encoder, Dice + BCE loss function
Twin FNN	2469 → 512 → 256 → 128 → 1 (80/20 split, early stopping, patience = 20)

**Table 2:** Evaluation metrics and validation strategies for each data modality. Each pipeline was assessed using metrics appropriate to its task. For microbiome and transcriptome models, performance was evaluated via mean squared error (MSE), coefficient of determination (R^2^), and Pearson correlation (*r*) across 15 cross-validation folds. The imaging model was tested on a hold-out set using IoU, precision, recall, and Dice. The twin pipeline was validated on true labels using regression metrics and tested for microbiome heritability using a two-sample *t*-test.

Modality	Primary Metrics	Validation Protocol
Microbiome	MSE, R^2^, Pearson *r*	five-fold CV × 3 repeats
Transcriptome	MSE, R^2^, Pearson *r*	five-fold CV × 3 repeats
Imaging	IoU, precision, recall, Dice coefficient	20-image hold-out validation
Twin Cohort	MSE, R^2^, Pearson *r* (FNN); Bray–Curtis *t*-test	80/20 train–validation split

**Table 3:** U-Net segmentation metrics during training and on the 20-image validation set. Values in parentheses are 95% bootstrap confidence intervals (1000 resamples, random_state = 42).

Epoch	IoU	Precision	Recall
1	0.42	0.59	0.38
5	0.51	0.68	0.48
10	0.54	0.71	0.51
15	0.56	0.73	0.54
20	0.57	0.74	0.55
25	0.58	0.75	0.56
Summary (20-image hold-out, sigmoid = 0.50)	0.564 (95% CI 0.534–0.593)	0.624 (0.586–0.666)	0.877 (0.823–0.919)

**Table 4:** Summary of model performance across all pipelines. Microbiome and transcriptome pipelines show stable, low-error convergence under synthetic targets but no predictive power. The imaging pipeline reaches moderate segmentation accuracy, and the twin-cohort model captures a weak microbiome signal correlated with caries burden.

Modality	Mean MSE	Mean R^2^	Mean r	Imaging IoU	Imaging Precision	Imaging Recall
Microbiome	0.0008	−0.15	−0.02	–	–	–
Transcriptome	0.0010	−0.18	−0.03	–	–	–
Twin-Cohort FNN	91.82	−0.03	0.253			
Imaging (U-Net)	–	–	–	0.564	0.624	0.877

**Table 5:** Comparison of machine-learning studies on caries or oral-health datasets. The present work is unique in (i) deploying fully reproducible pipelines across three modalities and a genetically informative twin cohort, and (ii) embedding SHAP explanations in all tabular models. Abbreviations: AUC, area under the curve; *r*, Pearson correlation; CRC, colorectal-cancer controls; DMFT/dmft, decayed–missing–filled teeth/surfaces; IoU, intersection-over-union.

Study (year)	Cohort Size & Data Set	Modalities/Cohort Alignment	ML Model & Interpretability	Headline Performance	Notes
Lee et al. 2018 [19]	3000 bite-wing radiographs (single centre)	Imaging only; no molecular data	ResNet-50 CNN; saliency maps not reported	AUC = 0.96 for proximal-caries detection	Benchmark deep-learning image study
Casalegno et al. 2019 [23]	1140 near-infra-red transillumination (NIRT) images	Imaging only	EfficientNet; Grad-CAM visualisation	Accuracy = 0.92 for enamel-lesion detection	Shows alternative imaging modality
Topçuoğlu et al. 2020 [21]	237 saliva 16S samples	Microbiome only (CRC vs. healthy)	XGBoost + SHAP feature ranking	AUC = 0.88	First microbiome study to apply SHAP
Divaris & Joshi 2020 [22] (ZOE 2.0)	>4000 children; saliva 16S + socio-demographics	Molecular + phenotype (aligned); no imaging	Random forest; permutation importance	AUC ≈ 0.80 for early-childhood caries	Largest paediatric microbiome cohort to date
Present study (2025)	81 plaque 16S, 247 gingival arrays, 100 panoramics (un-matched) + 198 twin saliva (matched)	Three unimodal pipelines + twin validation	U-Net, two FNNs, twin FNN; SHAP on all tabular models	IoU 0.564 (imaging); *r* 0.25 (twin FNN)	First fully reproducible workflow spanning imaging, microbiome, transcriptome and twin heritability

## Data Availability

The datasets analysed during the current study are publicly available from their original repositories, including GEO (GSE10334), Figshare, and the Kaggle “Panoramic Dental Caries” dataset. Processed data, trained model weights, and reproducible Colab notebooks are available upon reasonable request from the corresponding author.
